# Peripheral CD4^+^CD8^+^ double positive T cells: A potential marker to evaluate renal impairment susceptibility during systemic lupus erythematosus

**DOI:** 10.7555/JBR.36.20220094

**Published:** 2022-09-28

**Authors:** Kai Chang, Wanlin Na, Chenxia Liu, Hongxuan Xu, Yuan Liu, Yanyan Wang, Zhongyong Jiang

**Affiliations:** 1 Department of Medical Laboratory, The General Hospital of Western Theater Command, Chengdu, Sichuan 610083, China; 2 Institute of Microbiology, Sichuan Center for Disease Control and Prevention, Chengdu, Sichuan 610044, China; 3 Biology Teaching and Research Group, Chengdu Experimental Foreign Languages School, Chengdu, Sichuan 611134, China; 4 Department of Medical Laboratory, Affiliated Cancer Hospital of Chengdu Medical College, Chengdu Seventh People's Hospital, Chengdu, Sichuan 610231, China

**Keywords:** CD4^+^CD8^+^ double positive T cells, lupus nephritis, susceptibility, systemic lupus erythematosus

## Abstract

Lupus nephritis (LN) has a high incidence in systemic lupus erythematosus (SLE) patients, but there is a lack of sensitive predictive markers. The purpose of the study was to investigate the association between the CD4^+^CD8^+^ double positive T (DPT) lymphocytes and LN. The study included patients with SLE without renal impairment (SLE-NRI), LN, nephritic syndrome (NS), or nephritis. Peripheral blood lymphocyte subsets were analyzed by flow cytometry. Biochemical measurements were performed with peripheral blood in accordance with the recommendations proposed by the National Center for Clinical Laboratories. The proportions of DPT cells in the LN group were significantly higher than that in the SLE-NRI group (*t*=4.012, *P*<0.001), NS group (*t*=3.240, *P*=0.001), and nephritis group (*t*=2.57, *P*=0.011). In the LN group, the risk of renal impairment increased significantly in a DPT cells proportion-dependent manner. The risk of LN was 5.136 times (95% confidence interval, 2.115–12.473) higher in cases with a high proportion of DPT cells than those whose proportion of DPT cells within the normal range. These findings indicated that the proportion of DPT cells could be a potential marker to evaluate LN susceptibility, and the interference of NS and nephritis could be effectively excluded when assessing the risk of renal impairment during SLE with DPT cell proportion.

## Introduction

Systemic lupus erythematosus (SLE) is an autoimmune inflammatory disease usually characterized by multiple organ damage, in which various tissues and organs can be attacked by immune complex deposition and lymphocyte infiltration. Renal impairment is the most common in SLE patients. Lupus nephritis (LN) constitutes one of the main clinical challenges in the patients and is a cause of significant morbidity and mortality^[1]^. The cumulative incidence of LN is relatively high in Asians (55%) and Africans (51%) with SLE. The outcome of patients with an early diagnosed LN was significantly improved in recent years^[2]^, and thus early diagnosis and treatment of LN can greatly improve the prognosis of patients with SLE^[3]^. However, there is a lack of high sensitivity and specificity markers for early diagnosis of LN.

Peripheral CD4^+^CD8^+^ double positive T (DPT) lymphocytes are regarded as extrathymic cells, and these cells are rarely reported because of their small proportion in humans. In recent years, people pay more attention to DPT cells due to the improvement in detection technology. Studies have shown that increased proportions of peripheral DPT cells are observed in target organs of various autoimmune diseases and acute viral infections^[4–5]^. However, the role and repartition of extrathymic DPT cells remain largely uncharacterized^[6]^.

This study applied a retrospective analysis method to analyze the association between SLE without renal impairment (SLE-NRI) and LN to find the sensitive indicators that can effectively predict the occurrence of renal impairment in patients with SLE. We found that the proportion of DPT cells could be used for evaluating LN susceptibility.

## Subjects and methods

### Study design and participants

This study was conducted between 2017 and 2019 in the population covered by the General Hospital of Western Theater Command. Participants were selected using a simple random sampling scheme (random number table). Of the 395 participating patients with renal impairment or SLE, 79 were diagnosed with SLE-NRI, 100 with LN, 108 with nephritic syndrome (NS), and 108 with nephritis (including glomerulonephritis and pyelonephritis).

Diagnosis of SLE-NRI and LN was made according to the American College of Rheumatology diagnostic criteria, and the criteria mainly included cheek erythema, discoid erythema, light allergy, oral ulcers, arthritis, serositis, renal lesions, neuropathy, hematological lesions, immunological abnormalities, and antinuclear antibodies. Diagnosis of NS and nephritis was made according to the World Health Organization diagnostic criteria^[7–8]^. The inclusion criteria for the study were patients with a definite diagnosis of SLE-NRI, LN, NS, and nephritis, disease course >1 year, age range from 10 to 85 years, and no sex restriction. Patients with tumors, low immunity, heart disease, and pregnancy/lactation, or using antihistamines were excluded.

This study used a detailed questionnaire and physical examination, which include age, blood pressure, medical history, occupation, imaging examination, B-ultrasound, peripheral blood examination, immunology examination, *etc*. Blood samples for further biochemical analysis were also collected from all the included patients.

The study was approved by the Ethics Committee of The General Hospital of Western Theater Command. Informed consents were obtained from all the participants.

### Blood sample collection and measurements

A peripheral blood sample was collected from each participant using blood collection tubes with an inert gel and spray-dried K2EDTA (BD, USA). Blood collection tubes were centrifuged at 106.2 *g* for 10 minutes. The blood serum was used directly for biochemical and immunological detection, or stored at −80 °C until use. All biochemical testing reagents were purchased from Beijing Strong Biotechnologies, Inc. (China). All immunological testing reagents were purchased from EUROIMMUN Medical Diagnostics, Ltd. (China). Biochemical measurements, including total cholesterol (T-Cho), triglyceride (TG), high-density lipoprotein cholesterol (HDL-C), low-density lipoprotein cholesterol (LDL-C), lipoprotein(a) [Lp(a)], apolipoprotein A (ApoA), apolipoprotein B (ApoB), cystatin C (Cys-C), blood urea nitrogen (BUN), serum creatinine (SCr), creatinine clearance rate (CCR), uric acid (UA), microalbuminuria (MA), urine creatinine (UCr), fibrinogen (Fib), D-dimer, and high-sensitivity C-reactive protein (hsCRP), were performed under the recommendations proposed by National Center for Clinical Laboratories. Anti-Sm antibody and anti-dsDNA antibody were measured by enzyme linked immunosorbent assay (ELISA).

### Flow cytometry

**Figure 1:**
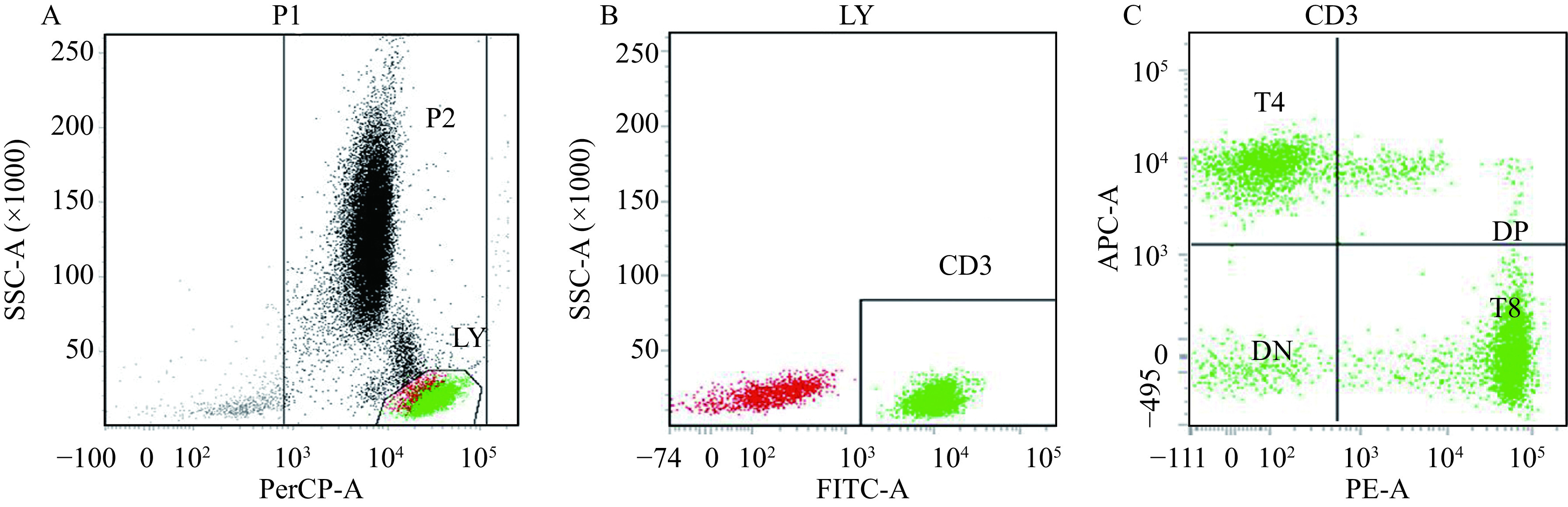
The maps of target lymphocytes and DPT cells by ﬂow cytometry.

Peripheral blood samples collected using blood collection tubes with spray-dried K2EDTA (BD, USA) were also used for flow cytometry. All biochemical testing reagents were purchased from BD Biosciences (USA). Peripheral blood lymphocyte subsets were analyzed on the BD FACSCanto Ⅱ Flow Cytometer (BD Biosciences). BD Multitest CD3 FITC/CD8 PE/CD45 PerCP/CD4 APC reagent (BD Biosciences) containing anti-CD3 fluorescein isothiocyanate, anti-CD8 phycoerythrin, anti-CD45 peridinin chlorophyll protein, and anti-CD4 allophycocyanin was used for identifying and determining the percentages and absolute counts of mature human T lymphocytes (CD3^+^), suppressor/cytotoxic (CD3^+^CD8^+^) T lymphocyte subsets, and helper/inducer (CD3^+^CD4^+^) T lymphocyte subsets in erythrocyte-lysed whole blood (***Fig. 1***). Briefly, the reagent cocktail (10 μL) was added to 50 μL EDTA-anticoagulated whole blood, and the sample was mixed and incubated for 30 min at room temperature in the dark. Erythrocytes were lysed by adding 500 μL of ammonium chloride hemolysis agent (BD Pharm Lyse, USA) for 15 min. Then the cells were washed, incubated with 2% paraformaldehyde in phosphate-buffered saline (PBS, pH 7.4), and measured on the flow cytometer^[9]^.

### Statistical analysis

The total clinical test results were collected, including peripheral blood lymphocyte subsets and biochemical measurements. Continuous variables were expressed as mean±SD and categorical variables were reported as counts and percentages. Analyses of Student's *t*-tests and chi-square tests were used to test for differences between groups for continuous and categorical variables, respectively. Multivariate logistic regression analysis was used to identify independent predictors of nephropathy^[10]^. Analyses were performed using SPSS version 19.0 statistical software (IBM, USA). A value of *P*<0.05 (two-sided) was considered statistically significant.

## Results

### Demographic data of the enrolled subjects

A total of 395 individuals were enrolled in this study, including 154 cases of males and 241 cases of females. Of all patients, 35% were aged <30 years, 48% aged range from 30 to 50 years, 15% aged from 50 to 70 years, and 2% aged >70 years.

### Test results and comparative analysis between the SLE and LN groups

Because of the high probability of LN in patients with SLE, we compared clinical profiles of the patients in the SLE-NRI and LN groups (***Table 1***). There was no significant difference in the proportions of sex and age between the SLE-NRI and LN groups. As compared with the SLE-NRI group, the levels of T-Cho, LDL-C, Lp(a), ApoB and Fib were significantly higher than that in the LN group in blood fatty acid and coagulative function detection. In liver and renal function biochemical examination, the levels of albumin to globulin ratio (A/G), BUN, SCr, Cys-C, UA, MA, and MA to UCr ratio (MA/UCr) were significantly higher in the LN group than that in the SLE-NRI group. In lymphocyte subsets examination, the proportion of CD3^+^CD4^+^CD8^+^ (DPT cells) was also significantly higher in the LN group than in the SLE-NRI group. There were no significant differences in levels of TG, HDL-C, ApoA, D-dimer, hsCRP, CD3^+^CD4^+^CD8^−^, CD3^+^CD4^−^CD8^+^, CD3^+^CD4^−^CD8^−^, and CD4^+^ to CD8^+^ T cells ratio (T4/T8) between the two groups. There was also no significant difference in the positive rate of anti-Sm antibody and anti-dsDNA antibody between the LN and SLE-NRI groups.

**Table 1:** Demographic and clinical characteristics of patients in the SLE-NRI and LN groups

Variables	SLE-NRI (*N*=79)	LN (*N*=100)	*t*/*χ*^2^ value	*P*-value
Male [*n* (%)]	11 (13.92)	13 (13.00)	0.065	0.799
Age (years)	36.54±14.15	40.82±13.79	1.557	0.121
T-Cho (mmol/L)	4.31±1.21	5.07±1.55	3.285	0.001
TG (mmol/L)	1.77±2.22	1.96±0.96	0.703	0.483
HDL-C (mmol/L)	1.44±0.58	1.48±0.55	0.417	0.667
LDL-C (mmol/L)	2.29±0.82	2.79±1.13	2.958	0.004
Lp(a) (mg/L)	170.81±153.08	294.15±310.48	2.601	0.011
ApoA (g/L)	1.30±0.36	1.34±0.37	0.454	0.651
ApoB (g/L)	0.76±0.30	0.90±0.36	2.343	0.021
A/G	1.71±0.38	1.51±0.44	−5.638	<0.001
BUN (mmol/L)	4.99±1.57	10.16±8.48	5.303	<0.001
SCr (μmol/L)	69.63±23.84	135.21±140.41	4.076	<0.001
Cys-C (mg/L)	0.91±0.26	1.87±1.22	6.674	<0.001
CCR (mL/minute)	95.09±19.61	64.85±46.88	−5.267	<0.001
UA (μmol/L)	305.15±118.00	402.37±162.85	4.421	<0.001
MA (mg/L)	29.04±68.60	1819.57±3357.35	4.092	<0.001
MA/UCr (mg/g)	38.44±110.04	2400.72±5912.72	3.066	0.003
Fib (g/L)	2.87±0.98	3.40±1.23	2.717	0.007
D-dimer (mg/L)	1.85±1.79	3.22±6.50	1.159	0.250
hsCRP (mg/L)	0.99±0.98	1.45±2.30	1.617	0.108
Anti-Sm antibody positive [*n* (%)]	20 (25.32)	27 (27.00)	0.081	0.776
Anti-dsDNA antibody positive [*n* (%)]	24 (30.38)	31 (31.00)	0.001	0.983
CD3^+^CD4^+^CD8^−^ (%)	32.59±9.92	32.15±8.89	−0.315	0.753
CD3^+^CD4^−^CD8^+^ (%)	33.69±11.95	37.00±10.50	1.968	0.051
CD3^+^CD4^+^CD8^+^ (%)	0.40±0.35	0.84±0.91	4.012	<0.001
CD3^+^CD4^−^CD8^−^ (%)	3.59±2.85	3.55±2.45	−0.096	0.924
T4/T8	1.18±0.77	0.99±0.60	−1.770	0.078
Data are presented as mean±SD, except for the data on gender and anti-Sm antibody and anti-dsDNA antibody positive rate. SLE-NRI: systemic lupus erythematosus without renal impairment; LN: lupus nephritis; T-Cho: total cholesterol; TG: triglyceride; HDL-C: high-density lipoprotein cholesterol; LDL-C: low-density lipoprotein cholesterol; Lp(a): lipoprotein(a); ApoA: apolipoprotein A; ApoB: apolipoprotein B; A/G: albumin/globulin; BUN: blood urea nitrogen; SCr: serum creatinine; Cys-C: cystatin C; CCR: creatinine clearance rate; UA: uric acid; MA: microalbuminuria; MA/UCr: MA/urine creatinine; Fib: fibrinogen; hsCRP: high-sensitivity C-reactive protein; T4/T8: CD4^+^ to CD8^+^ T cells ratio.				

### The proportion of CD4^+^CD8^+^ DPT cells was a risk factor for nephropathy in SLE patients

Multivariate logistic regression analysis was applied to analyze risk factors between the 79 SLE-NRI and 100 LN patients. As shown in ***Table 2***, the proportion of CD4^+^CD8^+^ DPT cells was an independent risk factor for nephropathy in SLE patients (odds ratio [OR], 5.136; 95% confidence interval [CI], 2.115–12.473; *P*<0.001). Hyperuricemia (OR, 3.285; 95% CI, 1.597–6.757; *P*=0.001) and hypertriglyceridemia (OR, 2.617; 95% CI, 1.288–5.577; *P*=0.013) may also contribute to the deterioration of LN in patients with SLE-NRI. No significant associations between any of the other factors (hypercholesterolemia or sex) and nephropathy were observed in SLE patients.

**Table 2:** Multivariate logistic regression analysis of independent risk factor for renal impairment in systemic lupus erythematosus

Variables	95% CI	*P*-value	OR value
Hypercholesterolemia	0.505–3.742	0.534	1.375
Hypertriglyceridemia	1.288–5.577	0.013	2.617
Hyperuricemia	1.597–6.757	0.001	3.285
Sex^a^	0.264–2.065	0.563	0.738
CD4^+^CD8^+^ DPT	2.115–12.473	<0.001	5.136
Hypercholesterolemia was diagnosed when serum total cholesterol >6.2 mmol/L. Hyperuricemia was diagnosed when serum uric acid >420 μmol/L in men or >360 μmol/L in women.Blood triglyceride levels were greater than 1.7 mmol/L in hypertriglyceridemia. The cut-off value of the proportion of CD4^+^CD8^+^ double positive T (DPT) lymphocytes was 1.42%. ^a^Male was used as the reference category for sex. CI: confidence interval; OR: odds ratio.			

### The proportion of CD4^+^CD8^+^ DPT cells in the LN group was significantly higher than that in the NS group

To further determine the impact of CD4^+^CD8^+^ DPT cells on the development of LN, we compared the test results between the NS and LN groups (***Table 3***). As compared with the NS group, the levels of HDL-C, LDL-C, ApoA, ApoB, and Fib were significantly lower in the LN group in serum lipid and coagulative function detection. In liver and renal function biochemical examination, the level of A/G was higher in the LN group than that in the NS group, but the levels of hsCRP and MA were higher in the NS group instead. In lymphocyte subset examination, the proportion of CD3^+^CD4^−^CD8^+^ and CD3^+^CD4^+^CD8^+^ T cells was also significantly higher in the LN group than that in the NS group. The T4/T8 ratio was significantly reduced in the LN group. The positive rate of anti-Sm antibody and anti-dsDNA antibody were significantly higher in the LN group than in the NS group. There were no significant differences between the two groups in the levels of T-Cho, TG, Lp(a), BUN, SCr, Cys-C, CCR, UA, MA/UCr, D-dimer, hs-CRP, CD3^+^CD4^+^CD8^−^, and CD3^+^CD4^−^CD8^−^ T cells.

**Table 3:** Demographic and clinical characteristics of patients in the NS and LN groups

Variables	NS (*N*=108)	LN (*N*=100)	*t*/*χ*^2^ value	*P*-value
Male [*n* (%)]	60 (55.56)	13 (13.00)	84.200	<0.001
Age (years)	46.45±18.68	40.82±13.79	−2.895	0.004
T-Cho (mmol/L)	7.21±3.04	5.07±1.55	−0.631	0.529
TG (mmol/L)	2.48±1.62	1.96±0.96	1.336	0.183
HDL-C (mmol/L)	1.81±0.73	1.48±0.55	−3.250	0.001
LDL-C (mmol/L)	4.18±2.20	2.79±1.13	−4.82	<0.001
Lp(a) (mg/L)	410.88±392.15	294.15±310.48	−1.755	0.081
ApoA (g/L)	1.57±0.63	1.34±0.37	−2.608	0.010
ApoB (g/L)	1.37±0.63	0.90±0.36	−5.182	<0.001
A/G	1.29±0.47	1.51±0.44	2.534	0.012
BUN (mmol/L)	8.91±6.20	10.16±8.48	1.220	0.224
SCr (μmol/L)	109.37±90.96	135.21±140.41	1.582	0.115
Cys-C (mg/L)	1.63±1.17	1.87±1.22	1.365	0.174
CCR (mL/minute)	66.95±26.19	64.85±46.88	−0.389	0.698
UA (μmol/L)	408.08±122.77	402.37±162.85	−0.285	0.776
MA (mg/L)	5212.46±7287.50	1819.57±3357.35	−3.793	<0.001
MA/UCr (mg/g)	3237.29±3670.86	2400.72±5912.72	−1.138	0.257
Fib (g/L)	4.18±1.64	3.40±1.23	−3.282	0.001
D-dimer (mg/L)	3.31±4.19	3.22±6.50	−0.092	0.927
hsCRP (mg/L)	21.15±57.17	1.45±2.30	−3.373	0.001
Anti-Sm antibody positive [*n* (%)]	1 (0.90)	27 (27.00)	27.046	<0.001
Anti-dsDNA antibody positive [*n* (%)]	2 (1.90)	31 (31.00)	32.346	<0.001
CD3^+^CD4^+^CD8^− ^(%)	34.18±10.47	32.15±8.89	−0.510	0.135
CD3^+^CD4^−^CD8^+ ^(%)	27.58±10.31	37.00±10.50	6.525	<0.001
CD3^+^CD4^+^CD8^+ ^(%)	0.53±0.36	0.84±0.91	3.240	0.001
CD3^+^CD4^−^CD8^− ^(%)	3.29±2.11	3.55±2.45	0.816	0.415
T4/T8	1.52±1.03	0.99±0.60	−4.446	<0.001
Data are presented as mean±SD, except for the data on gender and anti-Sm antibody and anti-dsDNA antibody positive rate. NS: nephritic syndrome; LN: lupus nephritis; T-Cho: total cholesterol; TG: triglyceride; HDL-C: high-density lipoprotein cholesterol; LDL-C: low-density lipoprotein cholesterol; Lp(a): lipoprotein(a); ApoA: apolipoprotein A; ApoB: apolipoprotein B; A/G: albumin/globulin; BUN: blood urea nitrogen; SCr: serum creatinine; Cys-C: cystatin C; CCR: creatinine clearance rate; UA: uric acid; MA: microalbuminuria; MA/UCr: MA/urine creatinine; Fib: fibrinogen; hsCRP: high-sensitivity C-reactive protein; T4/T8: CD4^+^ to CD8^+^ T cells ratio.				

### The proportion of CD4^+^CD8^+^ DPT cells in the LN group was significantly higher than that in the nephritis group

**Figure 2:**
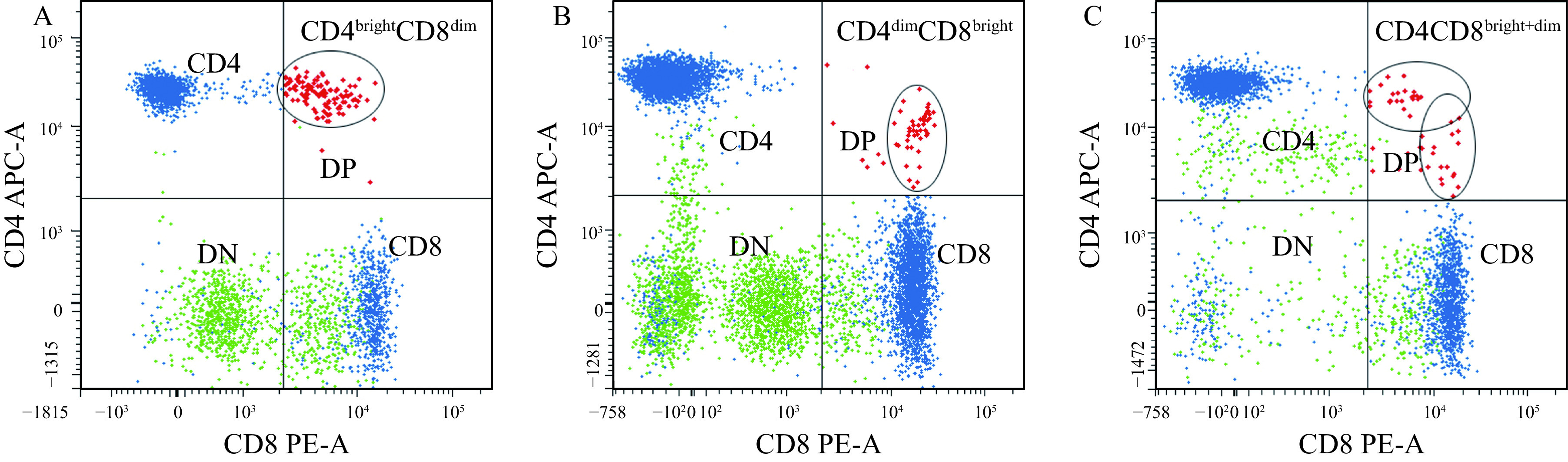
Flow cytometry maps of DPT cells with different fluorescence intensities.

To further determine the impact of CD4^+^CD8^+^ DPT cells on the development of LN, we compared the laboratory test results between the nephritis group and the LN group (***Table 4***). There was no significant difference in the proportions of age between these two groups. In serum lipid and coagulative function detection, there were no significant differences in levels of T-Cho, TG, HDL-C, LDL-C, Lp(a), ApoA, ApoB, A/G, Fib, D-dimer levels between the nephritis and LN groups. The level of BUN and Cys-C was higher in the LN group than that in the nephritis group, and the level of hsCRP was lower in the LN group than that in the nephritis group. The proportion of CD3^+^CD4^−^CD8^+^ T cells was significantly higher but the T4/T8 ratio was significantly reduced in the LN group, compared with that in the nephritis group. The positive rates of anti-Sm antibody and anti-dsDNA antibody were significantly higher in the LN group than those in the nephritis group. A high proportion (87%) of the patients in the LN group had CD4^bright^CD8^dim^ T cells, and a small proportion (4%) of the patients in this group had CD4^dim^CD8^bright^ T cells (***Fig. 2***). Meanwhile, CD4^dim^CD8^bright^ T cells were found in groups of SLE-NRI, NS, and nephritis. There were no significant differences between the two groups in the levels of age, T-Cho, TG, HDL-C, LDL-C, Lp(a), ApoA, ApoB, A/G, CCR, UA, MA, MA/UCr, Fib, D-dimer, and CD3^+^CD4^+^CD8 T cells (***Table 4***).

**Table 4:** Demographic and clinical characteristics of patients in the nephritis and LN groups

Variables	Nephritis (*N*=108)	LN (*N*=100)	*t*/*χ*^2^ value	*P*-value
Male [*n* (%)]	70 (64.82)	13 (13.00)	92.582	<0.001
Age (years)	40.85±16.46	40.82±13.79	−0.377	0.707
T-Cho (mmol/L)	4.57±1.41	5.07±1.55	1.630	0.106
TG (mmol/L)	1.64±0.98	1.96±0.96	1.613	0.110
HDL-C (mmol/L)	1.31±0.39	1.48±0.55	1.603	0.112
LDL-C (mmol/L)	2.60±1.06	2.79±1.13	0.856	0.394
Lp(a) (mg/L)	171.11±136.54	294.15±310.48	1.834	0.070
ApoA (g/L)	1.29±0.32	1.34±0.37	0.557	0.579
ApoB (g/L)	0.87±0.37	0.90±0.36	0.430	0.668
A/G	1.57±0.49	1.51±0.44	−0.668	0.505
BUN (mmol/L)	7.12±6.23	10.16±8.48	2.058	0.042
SCr (μmol/L)	123.38±139.95	135.21±140.41	0.446	0.657
Cys-C (mg/L)	1.28±0.61	1.87±1.22	2.763	0.007
CCR (mL/minute)	77.23±29.46	64.85±46.88	−1.477	0.142
UA (μmol/L)	354.91±124.35	402.37±162.85	1.654	0.100
MA (mg/L)	1083.72±1485.24	1819.57±3357.35	1.208	0.230
MA/UCr (mg/g)	897.33±1537.22	2400.72±5912.72	1.437	0.154
Fib (g/L)	3.57±1.56	3.40±1.23	−0.598	0.551
D-dimer (mg/L)	3.86±5.94	3.22±6.50	−0.352	0.726
hsCRP (mg/L)	19.24±35.04	1.45±2.30	−4.975	<0.001
Anti-Sm antibody positive [*n* (%)]	3 (2.80)	27 (27.00)	30.841	<0.001
Anti-dsDNA antibody positive [*n* (%)]	1 (0.90)	31 (31.00)	28.347	<0.001
CD3^+^CD4^+^CD8^− ^(%)	34.32±9.74	32.15±8.89	−1.267	0.207
CD3^+^CD4^−^CD8^+^ (%)	26.02±8.13	37.00±10.50	5.934	<0.001
CD3^+^CD4^+^CD8^+^ (%)	0.46±0.36	0.84±0.91	2.579	0.011
CD3^+^CD4^−^CD8^−^ (%)	4.76±3.99	3.55±2.45	−2.177	0.031
T4/T8	1.53±0.88	0.99±0.60	−4.137	<0.001
Data are presented as mean±SD, except for the data on gender and anti-Sm antibody and anti-dsDNA antibody positive rate. LN: lupus nephritis; T-Cho: total cholesterol; TG: triglyceride; HDL-C: high-density lipoprotein cholesterol; LDL-C: low-density lipoprotein cholesterol; Lp(a): lipoprotein(a); ApoA: apolipoprotein A; ApoB: apolipoprotein B; A/G: albumin/globulin; BUN: blood urea nitrogen; SCr: serum creatinine; Cys-C: cystatin C; CCR: creatinine clearance rate; UA: uric acid; MA: microalbuminuria; MA/UCr: MA/urine creatinine; Fib: fibrinogen; hsCRP: high-sensitivity C-reactive protein; T4/T8: CD4^+^ to CD8^+^ T cells ratio.				

### Biochemical and immunologic results in different CCR stages

**Figure 3:**
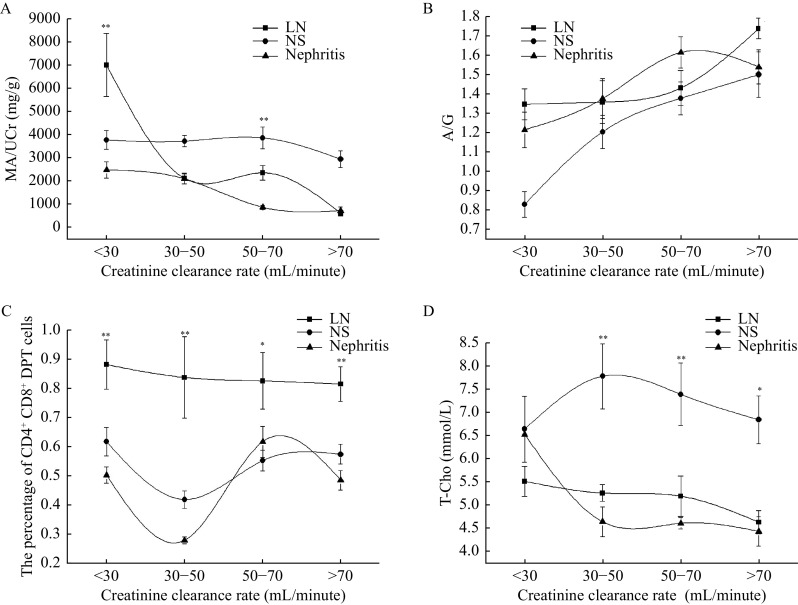
The trend chart of MA/UCr, A/G, CD4^+^CD8^+^ DPT cells, and T-Cho among the LN, NS, and nephritis groups.

To further illustrate the differences of the above-mentioned significant indexes in different renal impairment degree, the trend chart was plotted for the LN, NS, and nephritis groups in ***Fig. 3***. We analyzed differences in individuals' biochemical and immunologic results including MA/UCr, A/G, CD3^+^CD4^+^CD8^+^ cells (DPT cells), and T-Cho in different CCR stages. The MA/UCr values increased as CCR decreased. When CCR >30 mL/minute, the MA/UCr values of NS group were significantly higher than those in the LN and nephritis groups (***Fig. 3A***). The A/G values decreased as CCR decreased, and there was no significant difference among the three groups (***Fig. 3B***). The proportion of DPT cells in LN group was significantly higher than that in NS and nephritis groups (***Fig. 3C***). In addition, the proportion of DPT cells increased as CCR decreased. In the NS group, the levels of T-Cho were not significantly different in different CCR stages, but T-Cho levels in the NS group were significantly higher than those in the LN and nephritis groups, when CCR values over 30 mL/minute (***Fig. 3D***). These results indicate that MA/UCr and A/G were highly dependent on renal impairment. In the LN group, the proportions of DPT cells increased in a creatinine clearance rate-dependent manner. When CCR value ranges from 30 to 70 mL/minute, the T-Cho concentration in the NS group was significantly higher than in the LN and nephritis groups. Besides, the degree of renal impairment was positively correlated with the concentration of T-Cho.

### Comparative analysis and distribution map of CD4^+^CD8^+^ DPT cells in three groups

**Figure 4:**
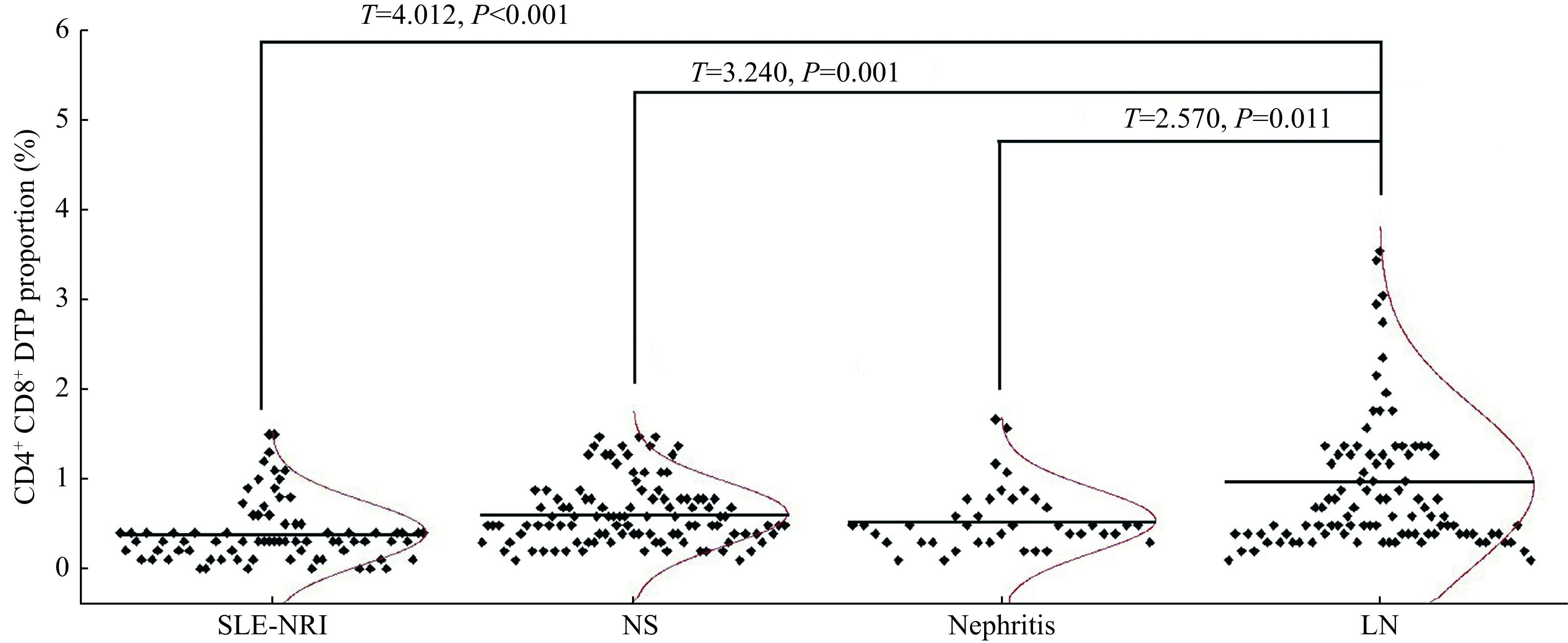
Comparative analysis and distribution map of CD4^+^CD8^+^ DPT in four groups.

There were significant differences between SLE-NRI and LN groups in the proportion of CD4^+^CD8^+ ^DPT cells (*t*=4.012, *P*<0.001). Compared with the NS and nephritis groups, the proportions of DPT cells were significantly higher in the LN group (*t*=3.240, *P*=0.001; *t*=2.57, *P*=0.011, respectively) (***Fig. 4***). The CD4^+^CD8^+ ^DTP proportion distribution width in the LN group was significantly greater than that in the SLE-NRI group, and the risk of renal impairment was significantly increased when the CD4^+^CD8^+ ^DTP proportion >0.84%. The mean value of the NS and nephritis groups was slightly higher than that of the SLE, but it did not affect the predictive value of the DTP for the LN.

## Discussion

SLE is an autoimmune disease with a worldwide distribution, always accompanied by the production of autoantibodies. LN remains a major cause of morbidity and mortality in patients with SLE^[11]^. Although the renal prognosis has been improved, major challenges remain in the management of disease progression and treatment of the disease due to the lack of early diagnosis method^[12]^.

Immature T cells expressing both CD4 and CD8 were traditionally thought to be the ancestor of T lymphocytes and to undergo thymic development^[13]^. After T cells had transitioned to naive T cells and exited the thymus, the CD4 or CD8 could no longer be expressed. Research has shown that healthy humans exhibit low proportions of CD4^+^CD8^+ ^DPT cells in peripheral blood^[14]^. At present, the origin and function of CD4^+^CD8^+ ^DPT cells are not clear. However, several mainstream views hold that CD4^+^CD8^+ ^DPT cells might trace back to immature CD4^+^CD8^+^ thymocytes, mature CD4^+^ single positive T cells, or mature CD8^+^ single positive T cells^[15]^. In the present study, we have found that an increase in the proportion of DPT cells is associated with renal impairment during SLE. The proportion of CD4^+^CD8^+ ^DPT cells in the LN group is significantly higher than those in both NS and nephritis groups. So, when assessing the risk of renal impairment during SLE with CD4^+^CD8^+ ^DPT cell proportion, we can effectively exclude the interference of NS and nephritis.

CD3^+^CD4^−^CD8^+^ T cells play important roles in innate and adaptive immune defense mechanisms protecting against both intrinsic and extrinsic factors, such as pathogens, viruses, and bacteria^[16]^. CD8^+^ T cells promote TNF-α, IL-6, and IFN-γ in responses to inflammation^[17]^. We observed that the CRP was significantly increased in both NS and nephritis groups (***Table 3*** and ***4***), which may be responsible for the increased CD3^+^CD4^−^CD8^+^T cell proportion. Since the difference in CRP was not significant in the LN group versus SLE group (***Table 1***), the CD3^+^CD4^−^CD8^+^ cells did not show a significant increases. As a subset of regulatory T cells, CD3^+^CD4^−^CD8^−^ T cells have therapeutic value for autoimmune disease, depending on their regulatory effects on CD8^+^ T cells, CD4^+^ T cells, and B cells^[18]^. CD4^+^ T cells have a dual function in peripheral T cells. First, it interacts with its ligand in an antigen-independent manner to induce contact between T cells and MHCⅡ expressing cells. Second, CD4 interacts with pMHCⅡ-TCR in an antigen-dependent manner to deliver Lck kinase to the complex and thus enhance T cell sensitivity^[19]^. However, we did not observed any significant differences in CD3^+^CD4^−^CD8^−^ cells and CD4^+^ T cells in all four groups.

In recent years, there has been more reported studies on DPT cells, and it has been found that DPT cells play an important role in the pathogenesis of autoimmune diseases and infectious diseases^[20]^. Wu *et al* have reported that the DPT cells play a key suppressive role in the production of autoantibodies in SLE^[21]^. In infectious diseases, DPT cells are a group of cells with a rapid response during acute HIV infection, which is different from the conventional T cell compartments^[22]^.

In our study, CD4^bright^CD8^dim^ was found to be the most common in patients with LN, and CD4^dim^CD8^bright^ was extremely rare. However, CD4^dim^CD8^bright^ was found in groups of SLE-NRI, NS, and nephritis. These results suggest that CD4^bright^CD8^dim^ DPT cells may be a potential biomarker in the development of renal impairment in SLE. Nevertheless, specific reasons and mechanisms need to be further studied.

The serum level of anti-Sm antibody is significantly correlated with the SLE disease activity index^[23]^. Monitoring of anti-Sm antibody titer may help assess the disease activity in SLE^[24]^. Clinical studies have found that anti-dsDNA antibody exhibits a significant correlation with SLE disease activity and multiple clinical manifestations^[25]^. However, the immunogenicity of dsDNA depends upon its context^[26]^. Therefore, the anti-dsDNA and anti-Sm antibodies do not directly reflect the SLE disease activity.

In conclusion, the significant finding of an association between LN and the proportion of DPT cells suggests that the proportion of DPT cells is related to LN susceptibility in SLE patients. A high proportion of DPT cells is an independent risk factor for LN, and the risk of LN is 5.136 times higher than the normal proportion in SLE patients. Therefore, the proportion of DPT cells is a potential marker to evaluate LN susceptibility in SLE patients. Furthermore, the deteriorative degree of nephropathy becomes more obvious as the proportion of DPT cells raised. On the basis of these results, we speculate that the occurence of DPT cells are a rapid response to the production of autoantibodies in SLE. Therefore, the increased proportion of DPT cells reflects the increase of autoantibodies, the severity of disease, and the destruction of renal tissue caused by immune complex.
